# Physiological and Therapeutical Action of Caffein

**Published:** 1868

**Authors:** 


### PHYSIOLOGICAL AND THERAPEUTICAL ACTION OF CAFFEIN.

The nunber of the *Archives de Physiologie et Pathologique* for January and February, 1868, contains an interesting paper on this subject by Dr. Leven. The following are the conclusions he draws from his experiments:

Caffein appears to directly stimulate the heart. When first absorbed, the circulation and respiration are accelerated, the pulse is more frequent and firmer, and the secretions more active.

The central nervous system, the brain and spinal cord, and the nerves are stimlated.

The musculor system of the life of relation and that of organic life contract violently.

The muscles of the former system are affected with trembling or with general contraction. The fibres of the stomach, of the intestines, and of the bladder also contract.

At a later period, after absorption of caffein, the action of the heart is lessened; the frequency and firmness of the pulse diminished; the muscular system becomes exhausted, but is not paralyzed. The nervous system also suffers exhaustion.

Caffein does not entirely extinguish reflex action, nor the functions of nerves and muscles.

It acts as a poison on different animals in different doses; it may be given to man in the dose of many grammes without injury.

It is readily eliminated from the system, and remains in it only a few hours.

He further states that caffein, like alcohol, diminishes the secretion of urea, but increases the quantity of urine excreted. It diminishes the waste of the organs, and economizes the tissues.

With two litres of coffee daily the Belgian miners undergo, without substantial food, excessive muscular exertion. The caravans which traverse the desert are supported by coffee during long journeys and lengthened privation of food. It is known that some old persons are almost exclusively nourished by coffee.—

